# Supplementary material for: Distinct checkpoint and homolog biorientation pathways regulate meiosis I in Drosophila oocytes
Source: bioRxiv. 2024 Aug 21:2024.08.21.608908. Preprint. [Version 1] doi: 10.1101/2024.08.21.608908 (PMC11370425; doi:10.1101/2024.08.21.608908)
Supplement: Supplement 1 [file NIHPP2024.08.21.608908v1-supplement-1.pdf]

bioRxiv preprint doi: https://doi.org/10.1101/2024.08.21.608908; this version posted August 21, 2024. The copyright holder for this preprint (which was not certified by peer review) is the author/funder, who has granted bioRxiv a license to display the preprint in perpetuity. It is made available under aCC-BY 4.0 International license.

Figure S 1. **Validation of *rod* and *mps1* reagents.**

(A) Wild-type oocyte with ROD^{GFP} in green, ZW10^{HA} in red, DNA in blue, and tubulin in white. Single channel images show ROD^{GFP} (middle) and ZW10^{HA} (right). (B) ROD^{GFP} localization in wild-type and *rod*^{RNAi} oocytes with ROD^{GFP} in green, DNA in blue, CENP-C in white, and tubulin in red. Single channel images (right) show ROD^{GFP}. (C) Quantification of ROD^{GFP} intensity at kinetochores, normalized to background GFP signal in wild-type and *rod*^{RNAi} oocytes (n = 142 and 126 kinetochores). Error bars show mean ± s.d.; ****P<0.0001 (unpaired two-tailed t test). (D) MPS1^{GFP} localization in wild-type and *Mps1*^{RNAi} oocytes with MPS1^{GFP} in green, DNA in blue, CENP-C in white, and tubulin in red. Single channel images (right) show MPS1^{GFP}. (E) Quantification of MPS1^{GFP} intensity at kinetochores, normalized to background GFP signal in wild-type and *Mps1*^{RNAi} oocytes (n = 75 and 71 kinetochores). Error bars show mean ± s.d.; ****P<0.0001 (unpaired two-tailed t test). All images are maximum intensity projections of z stacks. Scale bars represent 5 µm.

Figure S 2. **MAD1 and INCENP localization depends on SPC105R.**

Control (*Spc105R*^{B}) or mutant oocytes were incubated for one hour in 250 µM colchicine. (A) MAD1^{GFP} (green) localization in indicated genotypes, with DNA in blue, CENP-C in white, and tubulin in red. Single channel images (bottom) show MAD1^{GFP}. (B) Quantification of MAD1^{GFP} intensity at kinetochores, normalized to background GFP signal (from left to right, n = 91, 256, 224, 174, 212, 208, and 236 kinetochores). Error bars show mean ± s.d.; ****P<0.0001, ***P=0.0006 (unpaired two-tailed t test). (C) INCENP localization (green) in indicated genotypes with DNA in blue, CENP-C in white, and tubulin in red. Single channel images (bottom) show INCENP. (D) Quantification of INCENP presence at kinetochores (from left to right, n = 16, 29, 11, 31, 20, 18, 21, and 11 oocytes). Error bars show mean ± s.d.; ****P<0.0001, *P=0.02 (Fisher's exact test). All images are maximum intensity projections of z stacks. Scale bars represent 5 µm. All *Spc105R* mutants are in an *Spc105R*^{RNAi} background targeting the endogenous *Spc105R*.

Figure S 3. **Structure of SPC105R and mutant variants.**

A schematic of the *Spc105R* mutants used in this study. The coordinates on the schematic represent the first amino acid of each domain. The N-terminal includes SLRK and RISF. Following this is a domain with three MELT-like motifs, a region that contains two KI-like repeats, a central domain containing repeats with the consensus ExxEED, and the C-terminal region containing coiled-coil motifs.

Figure S 4. **MPS1 localization depends on SPC105R and NDC80.**

(A) MPS1^{GFP} localization in wild-type, *Spc105R*^{RNAi}, and *Ndc80*^{RNAi} oocytes, with MPS1^{GFP} in green, DNA in blue, CENP-C in white, and tubulin in red. Single channel images (bottom) show MPS1^{GFP}. (B) Quantification of MPS1^{GFP} intensity at kinetochores, normalized to background GFP signal in indicated oocytes (from left to

bioRxiv preprint doi: https://doi.org/10.1101/2024.08.21.608908; this version posted August 21, 2024. The copyright holder for this preprint (which was not certified by peer review) is the author/funder, who has granted bioRxiv a license to display the preprint in perpetuity. It is made available under aCC-BY 4.0 International license.

right, n = 389, 195, 139, 160, 165, 198, 128, and 143 kinetochores). Error bars show mean ± s.d.; ****P<0.0001 (unpaired two-tailed t test). (C) Quantification of MPS1^{GFP} intensity at kinetochores, normalized to background GFP signal in wild-type and *Ndc80*^{RNAi} oocytes (n=114 and 148 kinetochores). Error bars show mean ± s.d.; ****P<0.0001 (unpaired two-tailed t test). (D) MPS1^{GFP} localization in the indicated *Spc105R* mutants with MPS1^{GFP} in green, DNA in blue, CENP-C in white, and tubulin in red. All mutants are in an *Spc105R*^{RNAi} background targeting the endogenous *Spc105R*. Single channel images (bottom) show MPS1^{GFP}. All images are maximum intensity projections of z stacks. Scale bars represent 5 μm.

Figure S 5. **ROD and SPC105R localization in Z*w10-Spc105R*^{C} oocytes.**

(A) SPC105R localization in Z*w10-Spc105R*^{C}, either in the presence or absence of *Spc105R*^{RNAi}. SPC105R (green) was detected using an antibody which recognizes the N-terminal regions of SPC105R and does not detect SPC105R^{C}. DNA is in blue, CENP-C in white, and tubulin in red. All images are maximum intensity projections of z stacks. (B) ROD^{GFP} localization in oocytes expressing Z*w10-Spc105R*^{C}, either in the presence or absence of *Spc105R*^{RNAi}. ROD^{GFP} is in green, DNA in blue, CENP-C in white, and tubulin in red. Scale bars represent 5 μm.

Figure S 6. **Localization of Spindly variants and dependence of Spindly on ROD.**

(A) Examples of normal (left) and abnormal (right) ROD^{GFP} streaming in *Spindly*^{RNAi} oocytes, with ROD^{GFP} in green, DNA in blue, CENP-C in white, and tubulin in red. Single channel images (bottom) show ROD^{GFP}. Arrow points to abnormal streaming, which is defined as having ROD^{GFP} in the central region between the centromeres. (B) Spindly^{GFP} localization in wild-type or mutants of *Spindly*. Spindly^{GFP} is in green, DNA in blue, CENP-C in white, and tubulin in red. (C) Localization of Spindly$^{\Delta SB.GFP}$ in *rod*^{RNAi} oocytes, with Spindly^{GFP} in green, DNA in blue, CENP-C in white, and tubulin in red. Spindly^{GFP} and CENP-C are shown below the merged images. All images are maximum intensity projections of z stacks. Scale bars represent 5 μm. (D) Quantification of Spindly^{GFP} intensity at kinetochores in the indicated oocytes (from left to right, n = 123, 164, 83, and 75 kinetochores). Error bars show mean ± s.d.; ****P<0.0001 (unpaired two-tailed t test).
